# Ocular surface analysis and automatic non-invasive assessment of tear film breakup location, extension and progression in patients with glaucoma

**DOI:** 10.1186/s12886-019-1279-7

**Published:** 2020-01-06

**Authors:** Adriano Guarnieri, Elena Carnero, Anne-Marie Bleau, Nicolás López de Aguileta Castaño, Marcos Llorente Ortega, Javier Moreno-Montañés

**Affiliations:** 1 Department of Ophthalmology, Universidad de Navarra, Pamplona, Spain; 2 Medical Engineering Laboratory, Universidad de Navarra, Pamplona, Spain

**Keywords:** Keratograph 5 M, Tear film breakup, Dry area growth rate, BUT, NITBUT

## Abstract

**Background:**

Tear film stability is the key event in ocular surface diseases. The purpose of this study is to evaluate spatial and temporal progression of the tear film breakup using an automatic non-invasive device.

**Methods:**

Non-invasive tear breakup time (NITBUT) parameters, such as First NITBUT (F-NITBUT) and Average NITBUT (A-NITBUT), were evaluated in 132 glaucoma and 87 control eyes with the Keratograph 5 M device. Further analysis of this data was used to determine size, location and progression of tear film breakup with automatically identified breakup areas (BUA). The progression from First BUA (F-BUA) to total BUA (T-BUA) was expressed as Dry Area Growth Rate (DAGR). Differences between both groups were analysed using Student t-test for parametric data and Mann-Whitney U test for non-parametric data. Pearson’s correlation coefficient was used to assess the relationship between parametric variables and Spearman in the case of non-parametric variables.

**Results:**

F-NITBUT was 11.43 ± 7.83 s in the control group and 8.17 ± 5.73 in the glaucoma group (*P* = 0.010). A-NITBUT was 14.04 ± 7.21 and 11.82 ± 6.09 s in control and glaucoma groups, respectively (*P* = 0.028). F-BUA was higher in the glaucoma group than in the control group (2.73 and 2.28; *P* = 0.022) and was more frequently located at the centre of the cornea in the glaucoma group (*P* = 0.039). T-BUA was also higher in the glaucoma group than in the control group (13.24 and 9.76%; *P* = 0.012) and the DAGR was steeper in the glaucoma group than in the control group (34.38° and 27.15°; *P* = 0.009).

**Conclusions:**

Shorter NITBUT values and bigger, more central tear film breakup locations were observed in the glaucoma group than in the control group. The DAGR indicates that tear film rupture is bigger and increases faster in glaucomatous eyes than in normal eyes.

## Background

The ocular surface and its individual components make up the protective barrier between the eye and the outside world. The tear film has a vital role in providing lubrication and protection to the ocular surface, as well as maintaining a smooth, refractive surface for optimal visual performance [1]. It is regularly challenged by the environment (e.g., low humidity, wind exposure, pollutants) as well as disease. Chronic use of topical eyedrops causes ocular surface alterations [2]. Many clinical manifestations associated with low-grade chronic inflammation have been described in patients using anti-glaucoma treatments [3, 4]. Recognition and treatment of ocular surface disease in glaucoma patients may improve patient quality of life and medication adherence, as well as glaucoma treatment outcomes [5].

Much of our understanding of tear film dynamics comes from studies of tear film behaviour during the blink and the blink interval. Tear instability has been associated with tear deficiency, reduced quantity and quality of the lipid layer, altered tear composition, ocular surface irregularities, and ocular surface inflammation [6]. Tear film breakup time (TFBUT), the time from the completion of a blink to the appearance of the first dry spot on the cornea, defines the stability of the tear film and the integrity of the ocular surface, and has been a standard diagnostic test for many decades. Standard TFBUT analysis requires sodium fluorescein dye instillation into the tears and observation with a slit-lamp microscope using a cobalt blue light and a yellow filter. Acquiring a TFBUT is a relatively simple task, but interpreting the result is not straightforward because of its inherent variability. Despite its widespread use in both clinical and research settings, it is recognized that TFBUT has poor accuracy and reproducibility, as the peak yellow-green fluorescence is concentration and pH dependent [7]. A few approaches to improving repeatability have been suggested including taking multiple readings and minimizing the amount of fluorescein instilled [1]. Eliminating the use of fluorescein provides a non-invasive tear breakup time (NITBUT) value, which in theory represents a more physiological state of the tear film. In this case, tear film additives are avoided, and the examination environment should ideally introduce no additional sources of heat, air movement or intense light. The NITBUT recorded with automated systems was initially reported to be shorter than conventional TFBUT measurement, but other studies reported the reverse finding [8, 9].

In this study, we examine the spatial and temporal progression of the NITBUT with an automated system among glaucoma patients and normal subjects, to better understand and analyse the stability of the tear film.

## Methods

### Study design

We performed a cross-sectional study of patients with glaucoma, including 132 randomly selected eyes (right or left) of 132 patients using glaucoma medications for at least 1 year. Patients receiving certain medications such as anti-inflammatory or immunosuppressant drugs, or patients with previous eye surgery, were excluded from the study. The control group included 87 eyes from 87 asymptomatic people, who were consecutively recruited among hospital staff, nurses, patient relatives, and patients referred for a routine visual acuity examination without ocular diseases who had an intraocular pressure (IOP) of 20 mmHg or lower, normal visual fields, and no familial glaucoma history. Normal participants with dry eye symptoms or who were using any eyedrop, were excluded. All participants were European Caucasians and had no corneal or retinal pathology, no substantial media opacity that obscured the eye fundus, no history of amblyopia, no contraindication to dilation or intolerance to topical anaesthetic or mydriatic agents or fluorescein dye.

### Procedures

**Fig. 1:**
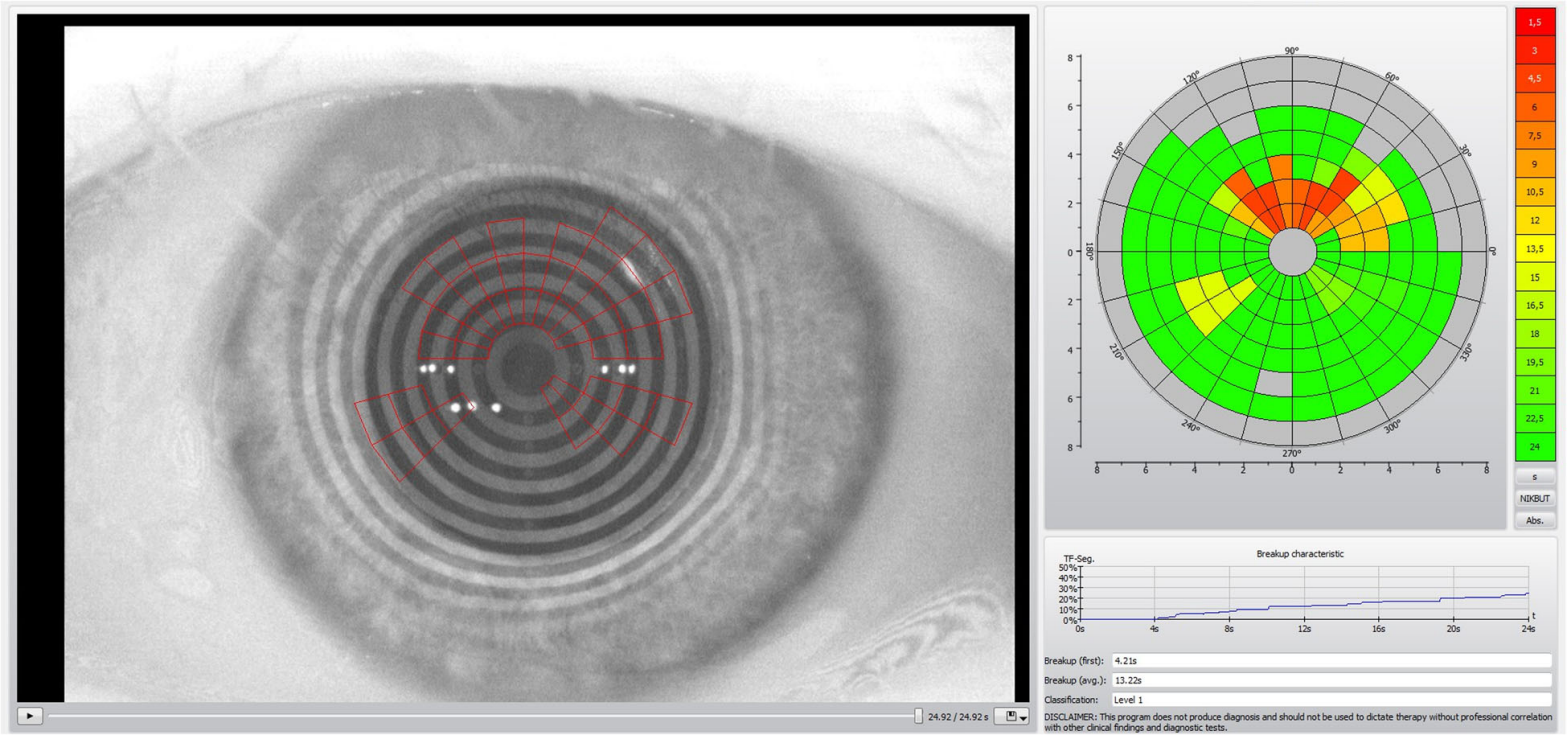
Keratograph 5 M outputs of NITBUT exam. On the left side, a corneal image with Placido disk projection and its disruptions (red squares) at the end of the measurement. On the upper right side, a color-coded map with the breakup locations and time. On the lower right side, a graph with temporal and spatial progression of the breakup characteristics. Breakup (first and average, or F-NITBUT and A-NITBUT) values are displayed

At the beginning of the visit, an ocular surface analysis using the Keratograph 5 M software version 2.5r18 (Oculus Optikgerate GmbH, Wetzlar, Germany) was performed without fluorescein dye. Two experienced examiners (AMB and EC) performed all non-invasive evaluations. All the measurements were taken between 10:00 a.m. and 6:00 p.m. in one single visit in a dimly lit room with controlled temperature (21–24 °C) and humidity (30–60%). The software determined the NITBUT after alignment of the instrument head with the pupillary centre and after the subjects were asked to blink twice, using an infrared light (Fig. 1). Two measurements were performed and if the values were similar, the first measure was recorded; if the difference between both measures of the same eye exceeded 2 s, a third measure was performed; then the first of the two measures closer to the third one was recorded. After acquisition, two parameters were recorded; the First NITBUT (F-NITBUT) defined as the time, in seconds, between the last complete blink and the first perturbation or irregularity of the 22 concentric rings of the Placido disc reflected on the corneal surface, and the Average NITBUT (A-NITBUT) defined as the average of all tear film breakups occurring in the measured period of up to 24.98 s (time limit set by the device’s software). The device has a minimal measure time of about 4 s to record NITBUT values. If the patient blinked after a brief period, before a value can be recorded, the examination was repeated. If the patient still was not able to keep the eye open for the minimal time of 4 s, that eye was not considered for further data analysis and excluded from the study. While recording the time of NITBUT values, the location of each rupture is also recorded by 168 small areas, or breakup areas (BUA). A graph with both measures and the areas of tear breakup are displayed (Fig. 1).

The number of simultaneous BUA at the F-NITBUT is referred as first breakup areas (F-BUA), and the total number of breakup areas at the end of the NITBUT measure, is referred as total breakup areas (T-BUA). The T-BUA is expressed as the percentage of the total exposed area measured by the device in each eye. The steepness [expressed in grades] of the increase in BUA after the F-NITBUT; that is, progression from F-BUA to T-BUA; allows analysis of tear film breakup rates and is referred as Dry Area Growth Rate (DAGR), as previously described [10]. A custom program written in MATLAB (MathWorks, Natick, MA, USA) was used to analyse the data recorded by the Keratograph 5 M to further evaluate these previous parameters. We generate a matrix in which the columns represented the time in fractions of one hundredth of a second (therefore 2498 columns), and the rows represented the randomly selected eye from each patient. We traced all the breaking points of each patient in an orderly manner looking for how many areas were break-up in each instant of time. Thus, we obtained a vector per patient of each group. In each group, we calculated the average of break-up zones at different times and the confidence interval, obtaining two vectors, one for normal cases and another one for cases with glaucoma.

**Fig. 2:**
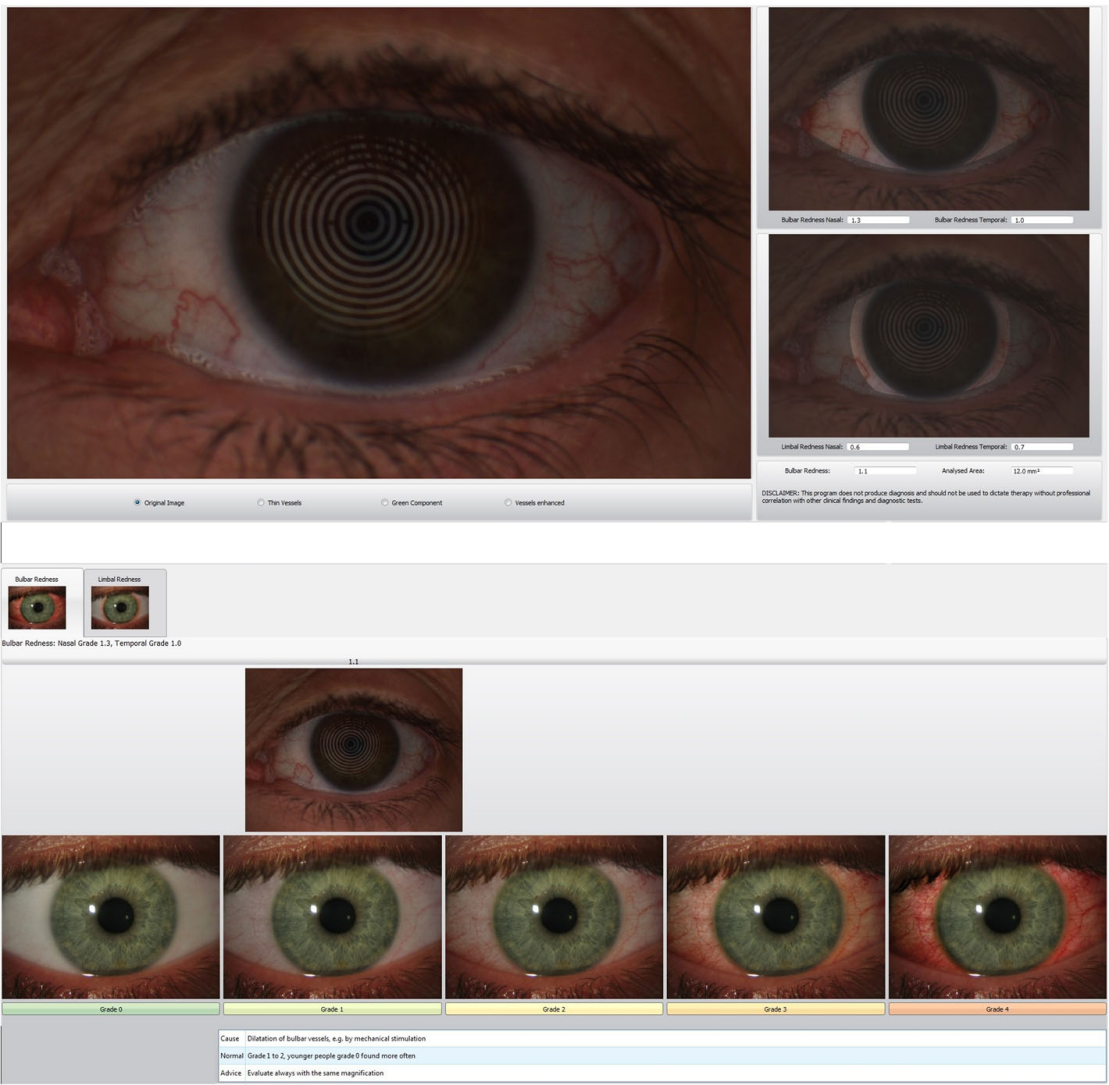
Keratograph 5 M output of Conjunctival Hyperaemia exam. On the image, a photograph of the eye with redness analysis on the right. On the lower image, conjunctival redness is automatically classified according to the Jenvis scale (grades 0–4)

The Keratograph 5 M was also used to quantify conjunctival hyperaemia (CH), evaluated after the NITBUT measure using a white light and automatically classified by the device software with the Jenvis grading scale from 0 to 4 using one decimal number (Fig. 2). The infrared camera of the device was also used to view the anatomy of the meibomian glands of the upper and lower eyelids. The status of the meibomian glands was evaluated with the Meiboscore [11], using a 0 to 3 scale (grade 0, no gland loss; grade 1, area of gland loss up to 33% of the total gland area; grade 2, area of gland loss between 33 and 66%; and grade 3, area of gland loss of 67% or more), and determined by hand tracing every picture of each eye with the ImageJ software (public domain, National Institutes of Health, USA).

Examiners (2 ophthalmologists), masked to the Keratograph 5 M results, performed ocular surface staining with 5 uL of 2% sodium fluorescein solution for TFBUT and for Oxford grading scale [12]. Schirmer test type 1 without anaesthesia was measured at the end of the visit.

### Statistical analysis

The Shapiro-Wilk test assessed the normality of the quantitative variables. Normally distributed variables were expressed as the mean and standard deviation (SD), and non-normally distributed variables were summarized using box plots. Differences between both groups were analysed using Student t-test for parametric data and Mann-Whitney U test for non-parametric data. Pearson’s correlation coefficient was used to assess the relationship between parametric variables and Spearman in the case of non-parametric variables. Data was evaluated using the SPSS program version 20.0.1 (SPSS Inc., Chicago, IL, USA).

## Results

### Demographics

The mean age in all patients (*n* = 219) was 67.65 ± 12.43 years (range 19–89); 66.95 ± 11.55 and 68.11 ± 13.01 years in the control and glaucoma groups, respectively, with no differences between groups (*P* = 0.209). In the glaucoma group (*n* = 132), the numbers of concomitant treatments were one in 31 eyes, two in 45 eyes, three in 46 eyes, and four in 10 eyes. Prostaglandin analogues (PG) were used in 102 eyes (alone or in combination); beta-blockers (BB) in 94 eyes; carbonic anhydrase inhibitors (CAI) in 66 eyes, and α2-adrenergics (AA) in 37 eyes. Thirty patients used one eye-drop a day, thirty-two used two daily, forty-four three drops daily, and thirty-one patients instilled four or more eye drops per day. Only 19 patients in our study were using just one medication instilled once daily.

### NITBUT

**Fig. 3:**
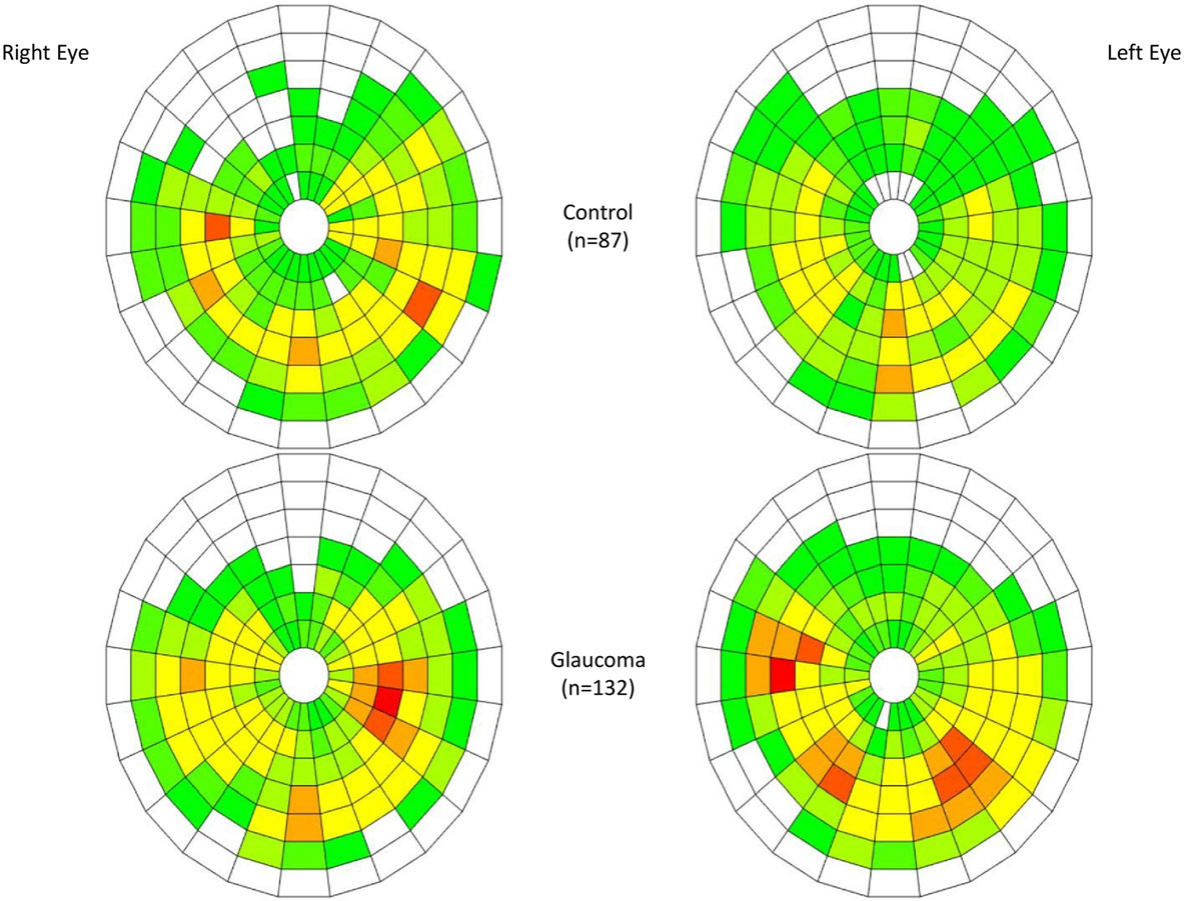
Spatial distribution of F-BUA. Comparison between both groups in a color-coded map with 168 small areas given by the Keratograph 5 M. In some eyes, several BUA broke simultaneously. Glaucoma patients showed more simultaneous BUA, that is, a bigger initial breakup area. Central location was more frequent in the glaucoma group. Scale from light green to red is represented as the frequency of F-NIBUT happening in each area. White areas: no breakup observed, either because no F-NITBUT was recorded in that area or because that area was not analysed by the device (e.g. shadows or premature blinking)

**Fig. 4:**
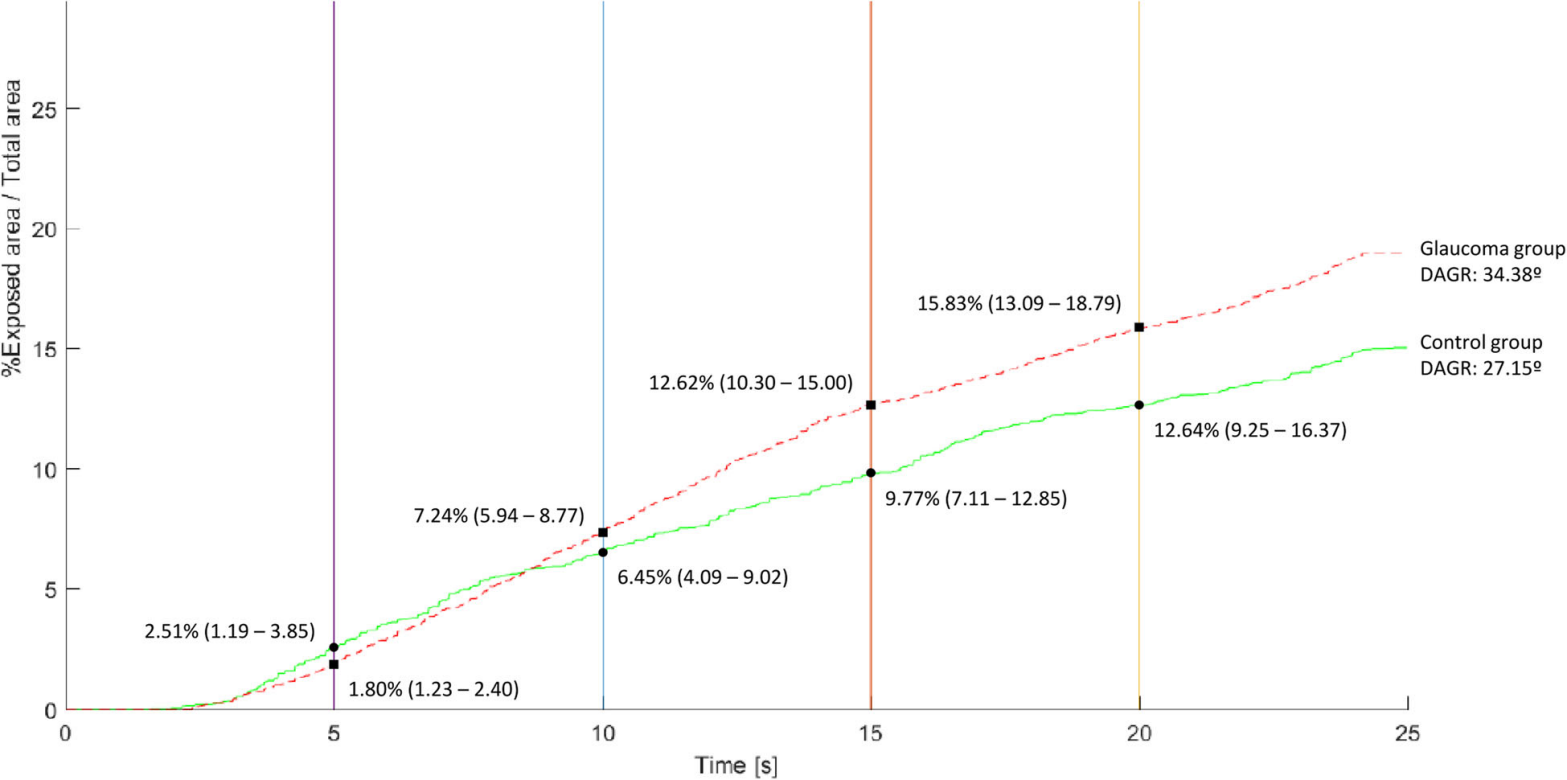
DAGR: Dry Area Growth Rate. NITBUT progression curves in each group. X axis: time in seconds of NITBUT. Y axis: Percentage of breakup areas according to total exposed area or area evaluated by the device. In each interval of time (5, 10, 15 and 20 s), the average breakup area of each group is displayed with a confidence interval of 95%

The mean F-NITBUT was 11.43 ± 7.83 s in the control group and 8.17 ± 5.73 in the glaucoma group, with statistically significant difference (*P* = 0.010). The A-NITBUT was also longer in the control group than in the glaucoma group (*P* = 0.028), with values of 14.04 ± 7.21 and 11.82 ± 6.09, respectively. The F-BUA (Fig. 3) was higher in the glaucoma group than in the control group (2.73 and 2.28; *P* = 0.022) and was more frequently located at the centre of the cornea in the glaucoma group than in the control group (*P* = 0.039). There were no differences in the peripheral corneal zones between both groups (*P* = 0.175). T-BUA was also higher in the glaucoma group than in the control group (13.24 and 9.76%; *P* = 0.012) and the DAGR (Fig. 4) was steeper in the glaucoma group than in the control group (34.38° and 27.15°; *P* = 0.009).

### Other Keratograph 5 M and invasive ocular surface evaluations

The average CH was 1.38 ± 0.40 in the control group and 1.72 ± 0.58 in the glaucoma group (*P* = 0.000), with statistically significant differences in all regions between both groups. Average Meiboscore was 1.10 ± 0.53 in the control group and 1.48 ± 0.53 in the glaucoma group (P = 0.000). TFBUT was 8.94 ± 4.77 s in the control group and 6.52 ± 4.29 in the glaucoma group, with statistically significant difference between both groups (*P* = 0.000); and it was correlated with F-NITBUT (r = 0.385; *P* = 0.000). All results are summarized in Table 1.

**Table 1:** Results

	Control group (*n* = 87)	Glaucoma group (*n* = 132)	*P* value
F-NITBUT (s)	11.43 ± 7.83	8.17 ± 5.73	0.010*
A-NITBUT (s)	14.04 ± 7.21	11.82 ± 6.09	0.028*
F-BUA (#)	2.28 ± 1.47	2.73 ± 1.45	0.022*
T-BUA (%)	9.76 ± 10.64	13.24 ± 12.14	0.012*
T-BUA at central cornea (%)	11.28 ± 14.83	15.13 ± 17.02	0.039*
T-BUA at peripheral cornea (%)	7.59 ± 8.17	9.16 ± 9.61	0.175
DAGR (°)	27.15 ± 23.18	34.38 ± 21.06	0.009*
Conjunctival Hyperaemia	1.38 ± 0.40	1.72 ± 0.58	0.000*
Nasal bulbar CH	1.32 ± 0.46	1.85 ± 0.61	0.001*
Nasal limbal CH	0.79 ± 0.37	1.13 ± 0.52	0.000*
Temporal bulbar CH	1.38 ± 0.46	1.52 ± 0.56	0.027*
Temporal limbal CH	0.88 ± 0.38	1.08 ± 0.50	0.000*
Meiboscore	1.10 ± 0.53	1.48 ± 0.53	0.000*
TFBUT (s)	8.94 ± 4.77	6.52 ± 4.29	0.000*
Oxford scale	0.44 ± 0.77	1.26 ± 0.98	0.000*
Schirmer test (mm/5 min)	13.46 ± 7.07	11.59 ± 6.78	0.044*

*F-NITBUT* First Non-Invasive Tear Breakup Time

*A-NITBUT* Average Non-Invasive Tear Breakup Time

*F-BUA* First Breakup Areas

*T-BUA* Total Breakup Areas

*DAGR* Dry Area Growth Rate

*CH* Conjunctival Hyperaemia

*TFBUT* Tear Fluorescein Breakup Time

* *P* value statistically significant if < 0.05

## Discussion

The Oculus Keratograph 5 M is an objective, non-invasive device that provides automatic and quantitative measurements of some ocular surface parameters, such as NITBUT and CH. It has been used to evaluate the ocular surface in different patient populations such as dry eye patients [13–18], Meibomian gland dysfunction [14, 17, 19], refractive surgery [20], and diabetes mellitus [21]. To our knowledge, only two articles [22, 23] from the same study group used the device to evaluate the ocular surface in glaucoma patients and did not find differences in NITBUT values between groups. We did find differences between glaucoma patients and healthy subjects in our study, not just in NITBUT values, but also in location and temporal progression of tear film breakups. To the best of our knowledge, this is the first time that the progression of the tear film breakup, as measured by the DAGR, is published in the literature using a non-invasive, objective and automatic approach.

The invasive nature of the TFBUT procedure can destabilize the tear film; either with the instillation of fluorescein drops, the heat emitted by the slit-lamp, or both. We expected TFBUT values to be shorter than NITBUT values, as other investigators have previously reported [8, 24]. There are different methods to evaluate the NITBUT, such as the TearScope (Keeler, Windsor, UK) which requires a white light that can also facilitate tear film evaporation or tear reflex. The Keratograph 5 M can use an infrared light that reduces photophobia and heat, minimizing tear film alteration during observation. As expected, we found longer NITBUT values than TFBUT values, with statistically significant correlation between them. In our study, patients with glaucoma had shorter tear film breakup time values (invasive or non-invasive) in average, than normal subjects.

We further analyse the NITBUT data to measure not only “when” the tear film breaks-up, but also “how” it does breakup, using location, size and progression rate. It is possible that the location and size of tear film breakup could affect dry eye symptoms. The central cornea is more sensitive than the periphery and this location should also affect vision because of tear film changes over the pupil and the visual axis, making the patient prone to premature blinking because of bad visual acuity. Other groups have reported F-NIBUT locations using the Keratograph 5 M device in other patients’ populations such as; inferonasal quadrant in meibomian gland disease [18], central location in post-LASIK patients [20], and supratemporal quadrant in healthy subjects [18]. In our study, we found the central location of BUA in patients with glaucoma more susceptible to tear film breakup when compared to controls, therefore, shorter TFBUT and NITBUT values in the glaucoma group were expected and observed. Quantitative analysis of the NITBUT demonstrates that the F-BUA is bigger in glaucoma patients than in controls and continues to rapidly destabilize leaving a bigger T-BUA before the blink. NITBUT analysis seems to be more reliable in disease than in healthy population [16], and this could explain our findings in the control group, such as bigger confidence interval of F-BUA at 5 s of NITBUT measure or different location of F-NITBUT comparing to previously reported [18].

This study had some limitations. It was an observational cross-sectional study, where it is not possible to determine how the longitudinal changes of ocular surface alterations under topical anti-glaucoma eye-drops are related to the Keratograph values. Also, it was impossible to evaluate the effect of each medication alone, since most of the glaucoma patients in our study were using at least 2 different concomitant medications. Moreover, a significant number of patients changed their medications several times after diagnosis and before current examination, therefore, there was a potential cumulative effect in those patients. Finally, we were unable to analyse the preservative effect in eyedrops, because only 5 patients in our glaucoma group were using non-preservative medication exclusively.

## Conclusions

The Keratograph 5 M may provide a simple, automatic, non-invasive assessment for ocular surface diseases. Determination of NITBUTs values along with its spatial and temporal progression may assist eye doctors in early treatment and prevention of further aggravation. For NITBUT to be more widely acceptable to clinicians, it is important for commercial developers to reveal underlying algorithms to allow for further analysis. As different breakup patterns can be caused by different mechanisms and not by a single primary event [25], automatic non-invasive devices should be able to recognize breakup patterns and future developments may include its detection to delineate specific pathology such as poor wettability or surface irregularities [26]. To our knowledge, this is the first study to automatically evaluate the location of tear film breakup in glaucoma patients and temporal progression of the tear film breakup using a non-invasive device. Future research involving prospective designs is needed to validate this potential biomarker in ocular surface disease.

## Data Availability

The datasets used and/or analysed during the current study are available from the corresponding author on reasonable request.

## Abbreviations

AA: Alfa2-adrenergics
A-NITBUT: Average Non-Invasive Tear Breakup Time
BB: Beta-blockers
BUA: Breakup Area
CAI: Carbonic Anhydrase Inhibitors
CH: Conjunctival Hyperaemia
DAGR: Dry Area Growth Rate
F-BUA: First Breakup Areas
F-NITBUT: First Non-Invasive Tear Breakup Time
IOP: Intraocular Pressure
NITBUT: Non-Invasive Tear Breakup Time
PG: Prostaglandin Analogues
T-BUA: Total Breakup Areas
TFBUT: Tear Fluorescein Breakup Time
